# Dietary patterns associated with hyperuricemia among the southeast coastal Chinese population

**DOI:** 10.3389/fnut.2025.1670666

**Published:** 2025-10-08

**Authors:** Til Bahadur Basnet, Qingling Su, Jiamin Gong, Xiaoyin Huang, Wanxin Li, Jun Chen, Ruimei Feng, Shanshan Du, Haomin Yang, Weimin Ye

**Affiliations:** ^1^Department of Epidemiology and Health Statistics, School of Public Health, Fujian Medical University, Fuzhou, China; ^2^Division of Quantitative and Clinical Sciences, Department of Obstetrics and Gynecology, Vanderbilt University Medical Center, Nashville, TN, United States; ^3^Institute of Population Medicine, School of Public Health, Fujian Medical University, Fuzhou, China; ^4^Department of Epidemiology, School of Public Health, Shanxi Medical University, Taiyuan, China; ^5^Department of Medical Epidemiology and Biostatistics, Karolinska Institutet, Stockholm, Sweden

**Keywords:** dietary pattern, cross-sectional study, animal food, principal component analysis, southeast coastal Chinese population

## Abstract

**Introduction:**

Various foods or food groups and nutrients are correlated with serum uric acid levels. However, the findings were not consistent across different populations, and the mechanisms remain unclear.

**Methods:**

In the baseline survey of the Fuqing Cohort, 4,326 participants were selected from Southeast coastal Chinese communities, and their dietary patterns were derived from a validated food frequency questionnaire using principal component analysis. Logistic regression was used to estimate the risk of hyperuricemia across the quintiles of each dietary pattern. Additionally, we performed mediation analysis to assess the potential mediating role of metabolic factors.

**Results:**

Based on the parallel analysis, four principal components were retained, explaining 46% of the total variance. Higher consumption of animal-based food (meat, fish, and seafood), bean products, sweets, desserts, and fried foods is significantly associated with an increased risk of hyperuricemia (Odds ratio for highest quintile of this dietary pattern vs. lowest: 1.36; 95% CI: 1.07, 1.72). Participants under 60 years showed a notably higher relative risk, which was significantly mediated by body mass index in combination with low-density lipoprotein and/or fatty liver.

**Conclusion:**

Greater adherence to a high-protein diet, along with fried food, sweets, and desserts, increases the risk of hyperuricemia, particularly in people under 60 years of age. Moderate consumption of an animal-based diet and significantly reducing the intake of sweets and fried foods may help prevent the risk of hyperuricemia.

## Introduction

Hyperuricemia is a main causal risk factor of gout, and its prevalence is also associated with cardiovascular diseases (CVDs), kidney disease, diabetes, and metabolic disorders (1–3). However, recent studies show a bidirectional relationship between hyperuricemia and these diseases (4). The global burden of hyperuricemia remains substantial, with both its incidence and prevalence continuing to rise. For instance, its prevalence rate was 20.1% in the USA (5), and the pooled prevalence of hyperuricemia was 16.4% (95% CI: 15.3–17.6%) in a recent meta-analysis of epidemiological studies among the Chinese population (6). Hyperuricemia prevalence is higher in men than in women, and it decreases in men with increasing age, while the opposite trend was observed in women (7).

Modifiable risk factors, including lifestyle and diet, are the main predictors of higher serum uric acid (SUA) (7–9). Although genetic factors play a vital role in elevating SUA levels, they cannot be modified (10). Due to the complexity of diet and the consumption of multiple food items at a time, nutrients from foods mostly correlate. A combination of some nutrients could synergistically influence disease risk. Therefore, the group of correlated food items as a single component can be derived from multivariate data analysis through a statistical technique, like principal component analysis (PCA), exploratory factor analysis, or reduced rank regression (11, 12). The above methods help determine the role of diets in predicting the risk of diseases, such as hyperuricemia, diabetes, cardiometabolic diseases, and kidney disease (13).

Previously, a purine-rich diet was considered a significant risk factor for high SUA (14, 15); however, later studies have revealed that a purine-rich diet derived from plant products is well-tolerated and does not substantially increase SUA (16). Higher consumption of animal products, mainly meat and seafood, is linked with increased SUA levels (17–19). Western diets characterized by high levels of saturated fat, processed meat, added sugar, and refined grains (20) have been associated with elevated SUA levels. A meta-analysis finds that the risk of hyperuricemia is positively correlated with the intake of red meat, seafood, alcohol, or fructose, and negatively correlated with the intake of dairy products, soy foods, and coffee drinks (21). In contrast, plant-based diets (16, 22), the Mediterranean diet (23), and the Dietary Approach to Stop Hypertension (DASH) diet (20, 24) have been shown to decrease the risk of hyperuricemia.

Over the past decade, research has focused on identifying dietary patterns and/or constructing hyperuricemia risk prediction models. However, available evidence has discrepancies in findings due to varied food consumption practices across different populations, different food group classifications, and small sample sizes. Therefore, the present study aims to identify dietary patterns using data-driven analysis and evaluate their association with the risk of hyperuricemia among the southeast coastal Chinese population.

## Methods

### Participants

The present study was a cross-sectional survey from the Fuqing Cohort Study, which began in July 2020, and a total of 7,662 individuals aged 35–75 years old from Gaoshan Town participated in the study till June 2021. The Fuqing Cohort Study was initiated to explore the natural history and risk factors of chronic non-communicable diseases, including cancer, diabetes, fatty liver, etc. The current analysis excluded the participants with self-reported chronic conditions, such as CVDs, chronic kidney and liver diseases, and cancer. Furthermore, self-reported or under-treatment cases of diabetes, hypertension, and hyperuricemia were excluded as these populations might have changed their dietary habit. After excluding subjects who met the above criteria, we included 4,326 participants for the final analysis (Supplementary Figure 1). Each participant was interviewed by a trained investigator using an electronic structured questionnaire^1^ that collected information on socio-demographic information, lifestyle and dietary intake habits, medical and medication history, and family history of the disease. The present study was approved by the ethical review committee of Fujian Medical University [2017–07 and 2020–58], and each participant provided written informed consent before participation in the study.

### Dietary assessment

Trained personnel administered a 93-item food frequency questionnaire (FFQ) to obtain the usual dietary intake over the past year. The method used to evaluate the validity and reliability of the FFQ is described in a prior Chinese study (25). Briefly, 24-h diet recalls were conducted over 4 days for 727 volunteers in our study, spanning four seasons from November 2020 to March 2022. During the final 24-h diet recall, these volunteers were also asked to complete a second FFQ. In general, for food groups, 62 to 84% of participants were categorized into the same quartile by repeated FFQs, which demonstrates a moderate to strong level of agreement. Moreover, validity assessments revealed moderate agreement (0.17–0.57) for most food groups between FFQs and 24-h dietary recalls. In the FFQ, the frequency of food consumption was recorded yearly, monthly, weekly, and daily. The amount per serving was recorded in the Chinese unit *Liang* (1 *Liang* = 50 grams). Low-consumption food items in daily life, such as candy and nuts, were measured in grams, while drinks or juice were measured in a bottle (500 mL). For each food item, the individual participant’s daily intake was calculated by the amount per time and the number of times per day. Finally, 93 food items were collapsed into 12 pre-defined food groups based on the similarity of foods in terms of nutrient content (Supplementary Table 1), which were used in further analysis. We did not include alcohol and any beverages in our food groups for analysis.

### Laboratory testing

Each serum sample was measured on an automatic biochemical analyzer (TBA-120FR, TOSHIBA, Japan) with reagents from DiaSys Co., Ltd. (Golzheim, Germany). SUA was measured using an enzymatic colorimetric test with the uricase-peroxidase method, and its concentration was measured in mg/dl (1 mg/dL = 59.48 mmol/L). Serum total cholesterol (TC) and triglycerides (TG) were measured using a chromatographic enzymic method in the analyzer. Low-density lipoprotein cholesterol (LDL-C) and high-density lipoprotein cholesterol (HDL-C) were measured using a homogeneous method. Serum creatinine was measured using a kinetic test.

### Definition of hyperuricemia

Hyperuricemia was defined as SUA concentration > 7.0 mg/dL (416.4 μmol/L) for men or > 6.0 mg/dL (356.9 μmol/L) for women (26).

### Definition and classification of covariates

Height was measured, to the nearest 0.1 cm, without shoes, and weight was measured with an electronic bulk composition meter (BC-601, TANITA Corporation, Japan), to the nearest 100 g, without shoes, and with light clothes. The body mass index (BMI) was calculated as weight (in kilograms) divided by standing height (in meters squared). Arterial blood pressure was measured with the subject in the sitting position and after at least 5 min at rest. Health workers took the participants’ blood pressure measurements from their right arm, relaxed and supported by a table with an electronic sphygmomanometer (OMRON, U30, Japan). Each participant was measured twice, and the average of the two measurements was used in the analysis. If the difference between the two measurements was >5 mmHg, the third measurement was conducted and calculated as the average of the two measurements with similar values. Smoking was categorized as never, previous smoker (quit smoking for at least half a year), and current smoker (smoking for more than half a year for at least 1 cigarette per day). Likewise, alcohol consumption was classified into never, previous user, and current user (at least once per week). Physical activities were measured in metabolic equivalents (METs) per day and then categorized into low, moderate, and vigorous based on the tertile of MET per day. Non-alcoholic fatty liver disease (NAFLD) was diagnosed using ultrasound images (ALOKA Prosound α7, Japan) and was divided into normal, mild, and moderate-to-severe. The estimated glomerular filtration rate was calculated using the serum creatinine value, based on the method from chronic disease epidemiology (27).

### Statistical analysis

Participants’ characteristics were summarized using mean and standard deviation for continuous variables and frequencies and percentages for categorical variables. The Kruskal–Wallis test was used to examine the statistical differences in continuous variables between participants with and without hyperuricemia, while the association between categorical variables was tested using the χ^2^-test. Multivariate analysis of food items by PCA with varimax rotation was performed to identify the dietary patterns. Scree plots based on parallel analysis suggest the number of dietary patterns to retain. Dietary patterns (varimax-rotated components) are retained if an eigenvalue based on real data is larger than the corresponding average eigenvalue from a set of random data matrices. Factor loadings are the correlation coefficients between the principal component and the food groups, indicating the significance of each food group. The higher the absolute value of the factor loadings, the stronger the correlation between the food groups and the principal components or factors (12). Consequently, the principal components or factors are named based on the food groups that meet the selection criteria for the factor loadings. Therefore, those food items with a loading factor greater than the absolute value of 0.30 were considered the main contributors to dietary patterns and representative of their characteristics. Logistic regression was performed to estimate the odds ratio (OR) and 95% confidence intervals (95% CI) for hyperuricemia associated with each dietary pattern. Furthermore, the scores of each significant dietary pattern were divided into quintiles. Sub-group and mediation analyses were performed to assess interactions and mediating effects, respectively. Sensitivity analyses were performed to ascertain the extent to which the findings deviated from the initial analysis when model parameters were altered. All reported *p*-values were based on two-sided tests at a significant level of 5%. R statistical software version 4.0.1 was used for all the statistical calculations.

## Results

### Dietary patterns

As shown in the Scree plot (Supplementary Figure 2), parallel analysis and the elbow method recommended that four components were appropriate for our dietary dataset. Through PCA with varimax orthogonal rotation, we obtained four dietary patterns, accounting for a total of 46% of the variance (Supplementary Table 2). A correlation coefficient of the individual food item greater than |0.30| was considered a significant contributing food item for that component. Among the four varimax rotated components, the first one had a high intake of vegetables, tubers, fruits, and fish and seafood. The second component was characterized by a high intake of meat, fish and seafood, bean products, sweets, desserts, and fried foods. Dietary pattern 3 was characterized by a lower intake of staple foods and higher consumption of milk and dairy products, eggs, and fruits, while the fourth pattern had a notably high intake of nuts and low intake of staple foods.

### Participants’ characteristics

The present study included 4,326 participants, and the prevalence of hyperuricemia was 33.9% (male: 41.5%, female: 29.9%). Table 1 describes participants’ characteristics according to hyperuricemia status. The median age of respondents was 59 years (Interquartile range: 52–66), and more than two-thirds were female participants (65.2%). Participants with a higher BMI, higher systolic blood pressure (SBP) and diastolic blood pressure (DBP), fatty liver disease, higher LDL, and a lower eGFR rate were more likely to have hyperuricemia. Similarly, current smokers and alcohol users had an increased probability of having hyperuricemia.

**Table 1:** Characteristics of participants by hyperuricemia status.

Variable	Total population (*N* = 4,326)	Hyperuricemia (No; *N* = 2,858)	Hyperuricemia (Yes; *N* = 1,468)	*P*-value^*^
Sex				<0.001
Male	1,505 (34.8%)	880 (30.8%)	625 (42.6%)	
Female	2,821 (65.2%)	1978 (69.2%)	843 (57.4%)	
Age (years)				0.623
<60	2,215 (51.2%)	1,471 (51.5%)	744 (50.7%)	
60+	2,111 (48.8%)	1,387 (48.5%)	724 (49.3%)	
Smoking				<0.001
Never	3,183 (73.6%)	2,168 (75.9%)	1,015 (69.3%)	
Previous smoker	413 (9.6%)	248 (8.7%)	165 (11.3%)	
Current smoker	726 (16.8%)	442 (15.5%)	284 (19.4%)	
Alcohol use				<0.001
Never	3,845 (88.9%)	2,584 (90.4%)	1,261 (86.0%)	
Previous	164 (3.8%)	96 (3.4%)	68 (4.6%)	
Current user	315 (7.3%)	177 (6.2%)	138 (9.4%)	
Fatty liver				<0.001
Mold	2,629 (62.9%)	1935 (69.6%)	694 (49.5%)	
Moderate	1,088 (26.0%)	631 (22.7%)	457 (32.6%)	
Severe	464 (11.1%)	214 (7.7%)	250 (17.8%)	
Median and interquartile range (Q1, Q3)				
Age (years)				0.022
	59 (52, 66)	59 (51, 66)	59 (53, 66)	
BMI (Kg/m^2^)				<0.001
	24.0 (21.9, 26.3)	23.5 (21.5, 25.7)	25.2 (23.0, 27.5)	
Physical activity (METs/day)				0.341
	10.0 (6.6, 16.0)	10.0 (6.5, 16.2)	10.0 (7.0, 15.9)	
Fasting glucose (mm/L)				0.044
	5.0 (4.6, 5.5)	5.0 (4.6, 5.5)	5.1 (4.6, 5.6)	
HbA1c (%)				0.002
	5.9 (5.7, 6.2)	5.9 (5.7, 6.2)	5.9 (5.7, 6.3)	
LDL (mm/L)				<0.001
	3.2 (2.7, 3.7)	3.1 (2.7, 3.7)	3.2 (2.8, 3.8)	
HDL (mm/L)				<0.001
	1.6 (1.4, 1.8)	1.6 (1.4, 1.9)	1.5 (1.3, 1.7)	
TG (mm/L)				<0.001
	1.1 (0.8, 1.6)	1.1 (0.8, 1.5)	1.3 (1.0, 1.9)	
SBP (mmHg)				<0.001
	133 (120, 148)	132 (119, 148)	135 (122, 150)	
DBP (mmHg)				<0.001
	85 (78, 92)	84 (77, 91)	87 (80, 94)	
eGFR (mL/min/1.72m^2^)				<0.001
	96.1 (89.3, 103.0)	97.4 (90.8, 104.0)	93.3 (84.4, 100.8)	
Energy intake (Kcal/day)				0.268
	1733 (1,379, 2,184)	1723 (1,372, 2,180)	1748 (1,394, 2,196)	

BMI, body mass index; METs, metabolic equivalents; Kg/m^2^, kilogram per meter square; mm/L, millimole per liter; HbA1c, glycated hemoglobulin; LDL, low-density lipoprotein; HDL, high density lipoprotein; TG, triglyceride; SBP, systolic blood pressure; mmHg, millimeter of mercury; DBP, diastolic blood pressure; eGFR, estimated glomerular filtration rate; Kcal/day, kilocalorie per day.

*P-value for categorical variables were from chi-square test and continuous variables from Kruskal-Wallis test.

### Dietary patterns and hyperuricemia risk

**Figure 1:**
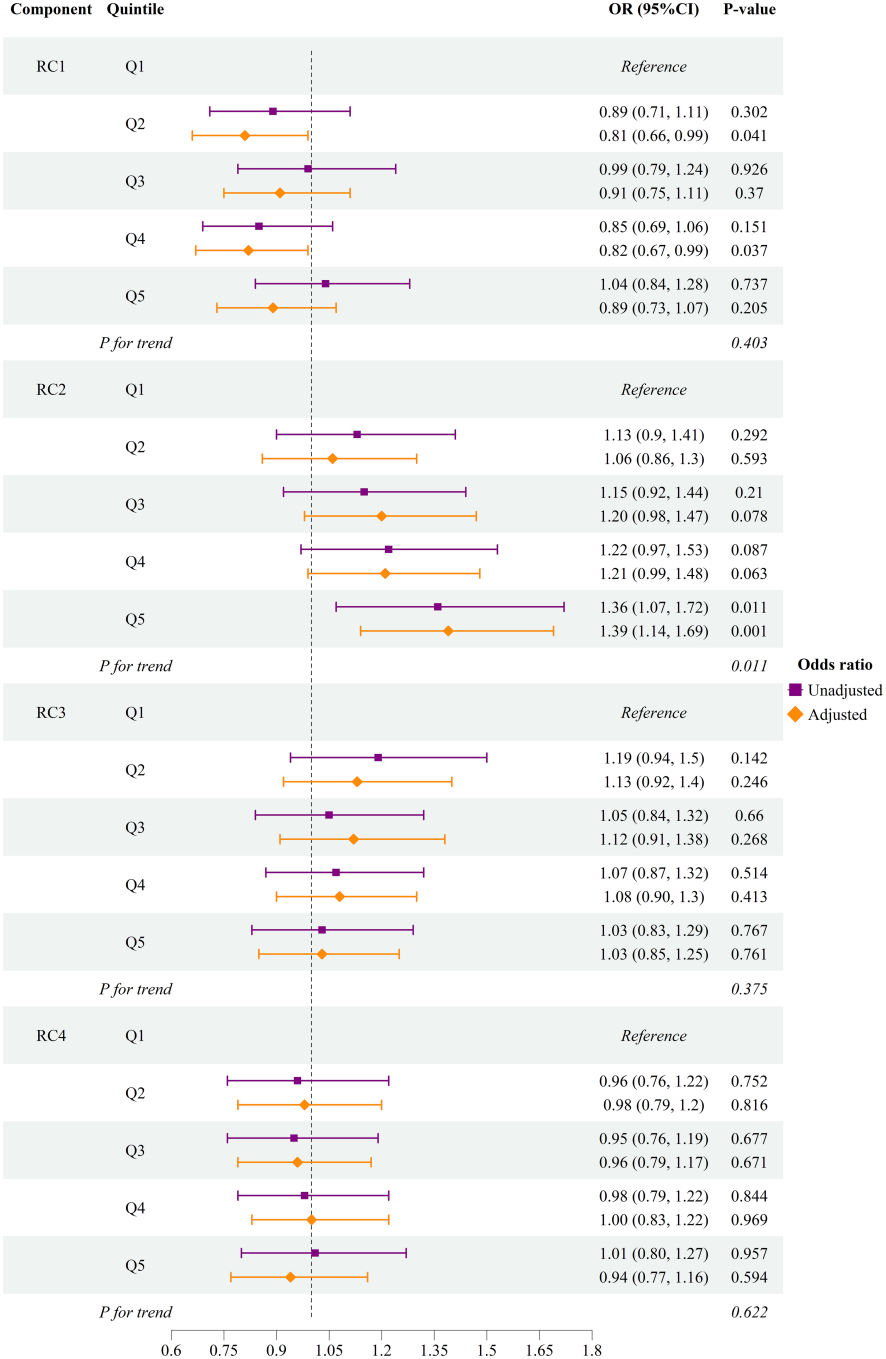
Odds of hyperuricemia by quintile of first four dietary components RC, rotated principal component; OR, odds ratio; CI, confidence interval. The adjusted model accounted for age, sex, body mass index, fatty liver disease, estimated glomerular filtration rate, alcohol user and smoking status, low-density lipoprotein, high-density lipoprotein, triglyceride, systolic blood pressure, diastolic blood pressure, fasting glucose, glycated hemoglobulin A1c, physical activity, total energy intake per day, and other rotated components (RC1, RC2, RC3, and RC4). RC1: The first component had a high intake of vegetables, tubers, fruits, fish, and seafood. RC2: The second component was characterized by a high intake of meat, fish and seafood, bean products, sweets, desserts, and fried food. RC3: The third component was characterized by a lower intake of staple food and higher consumption of milk and dairy products, eggs, and fruits. RC4: The Fourth pattern had a notably high intake of nuts and a low intake of staple foods. The forest plot illustrates the risk of hyperuricemia among individuals who were in the higher quintiles (Q2—Q5) of each PCA-derived dietary component, compared to those in the lowest quintile (Q1).

As shown in Figure 1, we developed two progressive models to estimate the relative risk of hyperuricemia for each PCA-derived dietary component. Out of four varimax-rotated principal components, only scores of the second component (RC2), where higher scores indicate a greater likelihood of following a dietary pattern comprising meat, fish and seafood, bean products, sweets, desserts, and fried food tries (“high-protein, high-fat, high-sugar” pattern), were significantly and directly associated with hyperuricemia. Those individuals who were in the fifth quintile, indicating the greater tendency to follow this dietary pattern, had a statistically significantly higher risk of hyperuricemia in the unadjusted [OR 1.39; 95%CI (1.14, 1.69); *p*-value = 0.001] and adjusted [AOR 1.36; 95%CI (1.07, 1.72); *p*-value = 0.011] models. The adjusted model accounted for age, sex, BMI, fatty liver disease, eGFR, alcohol use and smoking status, LDL, HDL, TG, SBP, DBP, fasting glucose, HbA1c, physical activity, total energy intake per day, and other rotated components (RC1, RC3, and RC4). We also modeled hyperuricemia as a function of each component’s scores (continuous variable) in the linear and non-linear models (Supplementary Tables 3–5 and Supplementary Figure 3). We observed an insignificant non-linear association, suggesting the association was linear only for the second component.

We further performed subgroup analysis stratified by age groups (<60 years vs. 60+ years) across the quintiles of the dietary pattern, which was significantly associated with the risk of hyperuricemia. In the age group under 60 years, individuals in the fifth quintile had a 60% higher risk of hyperuricemia compared to those in the first quintile. In contrast, among individuals 60 years or more, the risk increase was only 7% between the same quintiles. A significant interaction effect between age group (<60 years and 60+ years) and the dietary pattern quintile was observed in the fifth quintile (Table 2).

**Table 2:** Risk of hyperuricemia with quintiles of the “high-protein, high-fat, high-sugar” dietary pattern (the second rotated principal components).

Quintile	<60 years		60+ years		Interaction *P*-value
	AOR (95%CI)	*P*-value	AOR (95%CI)	*P*-value	
Q1	Reference		Reference		
Q2	0.98 (0.68, 1.41)	0.897	1.20 (0.90, 1.59)	0.213	0.051
Q3	1.12 (0.79, 1.60)	0.523	1.19 (0.89, 1.60)	0.240	0.942
Q4	1.39 (0.99, 1.97)	0.059	1.07 (0.78, 1.46)	0.693	0.098
Q5	1.60 (1.13, 2.28)	0.008	1.07 (0.75, 1.51)	0.719	0.019

RC, rotated component; OR, odds ratio; AOR, adjusted odds ratio; CI, confidence interval.

The adjusted model accounted for age, sex, body mass index, fatty liver disease, estimated glomerular filtration rate, alcohol user and smoking status, low-density lipoprotein, high-density lipoprotein, triglyceride, systolic blood pressure, diastolic blood pressure, fasting glucose, glycated hemoglobulin A1c, physical activity, total energy intake per day, and other rotated components (RC1, RC3, and RC4).

### Mediating analysis

Crucially, we performed the mediation analysis to evaluate the proportion of association between the dietary pattern (RC2) and hyperuricemia mediated by BMI, LDL, and fatty liver, both individually and in combination. The BMI is a crucial mediator in this relationship, and importantly, a higher mediating proportion was observed when it was combined with fatty liver and/or LDL. The proportions mediated were as follows: BMI and LDL: 13.2; (95%CI: 1.8, 48.1%); *p*-value = 0.022, BMI and fatty liver: 24.4%; (95%CI: 9.9, 67.3%); *p*-value 0.008, and BMI, LDL, and fatty liver: 27.8%; (95%CI: 24.4, 113%); *p*-value = 0.004 (Table 3).

**Table 3:** Individual and combined mediating role of fatty liver, body mass index, and low-density lipoprotein in the association between the second principal component and the risk of hyperuricemia in all populations, the <60 years, and 60+ years individuals.

Mediator’s parameter	Total		<60 years		60+ years	
	Estimate (95%CI)	*P*-value	Estimate 95%CI	*P*-value	Estimate 95%CI	*P*-value
BMI^*^						
Controlled direct effect	1.08 (1.01, 1.16)	0.020	1.14 (1.04, 1.24)	0.004	0.99 (0.89, 1.10)	0.860
Total natural direct effect	1.08 (1.01, 1.14)	0.020	1.13 (1.04, 1.21)	0.004	0.99 (0.90, 1.09)	0.860
Total natural indirect effect	1.01 (1.00, 1.02)	**0.044**	1.00 (0.99, 1.01)	0.376	1.02 (1.00, 1.03)	0.032
Total effect	1.09 (1.02, 1.16)	0.008	1.13 (1.05, 1.22)	0.004	1.01 (0.91, 1.11)	0.870
Proportion mediated (%)	10.0 (0.1, 40.9)	**0.048**	3.5 (−5.7, 15.7)	0.380	203 (−489, 392)	0.856
LDL^**^						
Controlled direct effect	1.08 (1.01, 1.17)	0.026	1.13 (1.05, 1.23)	0.004	0.99 (0.90, 1.10)	0.936
Total natural direct effect	1.08 (1.01, 1.16)	0.026	1.13 (1.05, 1.22)	0.004	0.99 (0.91, 1.10)	0.936
Total natural indirect effect	1.01 (1.00, 1.02)	**0.026**	1.01 (1.00, 1.02)	0.054	1.00 (0.99, 1.00)	0.954
Total effect	1.09 (1.02, 1.16)	0.008	1.13 (1.05, 1.23)	<0.001	0.99 (0.90, 1.10)	0.938
Proportion mediated (%)	11.0 (−0.7, 43.2)	0.058	6.3 (−0.1, 18.6)	0.054	−1.7 (−21.9, 21.9)	0.988
Fatty liver^***^						
Controlled direct effect	1.08 (1.01, 1.15)	0.026	1.13 (1.04, 1.23)	0.004	0.99 (0.89, 1.10)	0.848
Total natural direct effect	1.08 (1.01, 1.15)	0.026	1.12 (1.02, 1.21)	0.004	0.99 (0.89, 1.10)	0.848
Total natural indirect effect	1.00 (0.99, 1.01)	0.222	1.01 (0.99, 1.02)	0.456	1.00 (0.99, 1.01)	0.874
Total effect	1.08 (1.01, 1.15)	0.020	1.13 (1.04, 1.23)	0.004	0.99 (0.90, 1.10)	0.862
Proportion mediated (%)	6.1 (−6.1, 26.7)	0.234	4.9 (−8.6, 18)	0.454	−5.3 (−107, 100)	0.984
BMI and LDL^****^						
Controlled direct effect	1.08 (1.01, 1.16)	0.018	1.14 (1.04, 1.27)	0.008	0.99 (0.88, 1.10)	0.888
Total natural direct effect	1.08 (1.01, 1.14)	0.018	1.12 (1.03, 1.23)	0.008	0.99 (0.89, 1.09)	0.888
Total natural indirect effect	1.01 (1.00, 1.02)	**0.014**	1.01 (0.99, 1.03)	0.054	1.02 (0.99, 1.03)	0.064
Total effect	1.09 (1.02, 1.16)	0.006	1.14 (1.05, 1.24)	0.006	1.01 (0.91, 1.11)	0.878
Proportion mediated (%)	13.2 (1.8, 48.1)	**0.022**	10.1 (−0.3, 26.6)	0.058	217 (−502, 466)	0.864
LDL and Fatty liver ^*****^						
Controlled direct effect	1.08 (1.01, 1.15)	0.040	1.14 (1.05, 1.24)	0.002	0.99 (0.89, 1.10)	0.932
Total natural direct effect	1.08 (1.01, 1.14)	0.040	1.12 (1.05, 1.22)	0.002	0.99 (0.89, 1.10)	0.932
Total natural indirect effect	1.01 (0.99, 1.02)	0.114	1.01 (0.99, 1.03)	0.096	1.00 (0.99, 1.01)	0.892
Total effect	1.08 (1.01, 1.15)	0.028	1.14 (1.05, 1.23)	0.002	0.99 (0.89, 1.10)	0.946
Proportion mediated (%)	8.9 (−4.5, 42)	0.138	10.6 (−2.0, 27.5)	0.096	−1.2 (−141, 102)	0.984
Fatty liver and BMI^******^						
Controlled direct effect	1.09 (1.01, 1.16)	0.020	1.15 (1.05, 1.26)	0.004	0.99 (0.90, 1.10)	0.890
Total natural direct effect	1.08 (1.01, 1.14)	0.020	1.12 (1.04, 1.22)	0.004	0.99 (0.90, 1.10)	0.890
Total natural indirect effect	1.02 (1.01, 1.04)	**0.006**	1.02 (0.99, 1.04)	0.074	1.03 (0.92, 1.12)	0.024
Total effect	1.10 (1.03, 1.17)	0.002	1.14 (1.06, 1.25)	<0.001	1.02 (0.92, 1.12)	0.688
Proportion mediated (%)	24.4 (9.9, 67.3)	**0.008**	13.8 (−1.6, 36.3)	0.074	129 (−740, 734)	0.690
BMI, LDL, and Fatty liver						
Controlled direct effect	1.09 (1.01, 1.16)	0.032	1.15 (1.05, 1.27)	<0.001	0.99 (0.88, 1.12)	0.860
Total natural direct effect	1.08 (1.01, 1.14)	0.032	1.12 (1.04, 1.22)	<0.001	0.99 (0.89, 1.11)	0.860
Total natural indirect effect	1.03 (1.01, 1.04)	**<0.001**	1.03 (1.01, 1.05)	0.014	1.03 (1.00, 1.05)	0.016
Total effect	1.11 (1.03, 1.17)	0.004	1.16 (1.07, 1.26)	<0.001	1.02 (0.92, 1.13)	0.700
Proportion mediated (%)	27.8 (24.4, 113)	**0.004**	19.7 (4.6, 44.5)	0.014	124 (−618, 914)	0.700

CI, confidence interval; LDL, low-density lipoprotein; BMI, body mass index.

A bold value indicates a significant P-value for less than 0.05.

Adjusted for age, sex, hypertension, estimated glomerular filtration rate, smoking, alcohol drinking, log of physical activity.

Further adjusted for ^*^LDL and fatty liver; ^**^ BMI and fatty liver; ^***^LDL and fatty liver; ^****^fatty liver; ^*****^ BMI; ^******^ LDL.

## Discussion

In order to identify dietary patterns associated with hyperuricemia, we extracted four potential components using PCA of cross-sectional data (*N* = 4,326) from the Fuqing cohort study. Out of these four components, the one characterized by a higher intake of meat, fish and seafood, bean products, sweets, desserts, and fried foods was significantly associated with the risk of hyperuricemia. The dietary pattern is uniquely composed of high-protein food items: meat, fish, and seafood (animal-based diet), and bean products, along with sweets, desserts, and fried foods. We divided the scores of this dietary pattern into quintiles, with a higher quintile indicating greater adherence to the pattern. After adjusting for potential confounders, we observed a 36% increase in the odds of hyperuricemia among individuals in the highest quintile of this dietary pattern compared to those in the lowest quintile. Among people under 60 years of age, we found that those who followed a high-protein diet, along with sweets, desserts, and fried food, were more prone to hyperuricemia. Additionally, we explored that high BMI, either in combination with LDL or fatty liver, partially mediated this association.

The present study observed the direct association between hyperuricemia and the dietary pattern identified through PCA, characterized by higher eigenvector loadings for animal-based foods (meat, fish, and seafood), bean products, sweets, desserts, and fried foods. Congruent with our research, one previous study observed that animal-based food was associated with increasing hyperuricemia prevalence among Chinese people (18). Likewise, a propensity score-matched case–control study identified an animal-foods dietary pattern, which was rich in animal organs, seafood, and processed food, that elevated hyperuricemia risk among Chinese adults (17). Observational studies in the Western population also reported an increased hyperuricemia risk due to higher consumption of animal products rich in purine content, such as meat, sea fish, and other aquatic products (15, 19). Similarly, a cross-sectional analysis of food items and SUA levels in two Caucasian populations demonstrated that a higher intake of meat and eggs increased SUA levels (28). Importantly, although higher daily consumption of meat and seafood is associated with a higher SUA level, total protein intake is not linked to a higher SUA level in a nationally representative US adult population (15). The possible reason for increased SUA levels might be due to a higher amount of purine in meat, marine fish, and other aquatic products, which leads to increased SUA levels (29). Further study is suggested to gain insight into the amount of purine contained in commonly consumed sea fish and marine food among the Southeast coastal Chinese population. Understanding the genetic susceptibility to purine-rich diets in this population could help provide guidelines on the maximum intake of specific aquatic products to manage SUA levels.

In contrast, some previous observational studies reported non-significant findings regarding the association between total meat consumption and the risk of hyperuricemia among the Chinese population. For instance, a prospective study from Taiwan [31] and the Shanghai men’s study [32] could not establish a link between hyperuricemia risk and meat intake. The insignificant findings may be due to the diverse food intake culture among the Chinese population. Southeast coastal Chinese inhabitants have unique dietary cultures and customs. Traditionally, Chinese diets are composed of diverse foods, including both animal-based (eggs, meat, fish, and seafood) and plant-based foods (cereal, vegetables, fruits, nuts, pickles, and bean products) (30). However, a recent study shows the Chinese diet has been shifting away from the traditional diets toward high-fat, low-carbohydrate, and low-fiber diets. Nowadays, the nutrient intake among Chinese people has been deteriorating even more than that of the American people (31). Our study identified a dietary pattern characterized by high protein, high fat, and high sugar, which not only validated this alteration but also pointed to its association with hyperuricemia.

Several studies demonstrated that seafood, including other animal products, mainly red meat and purine-rich foods, adversely affects uric acid metabolism (17–19), indicating that seafood alone or in combination with other animal products may increase the risk of hyperuricemia. However, our dietary pattern had a higher loading of not only animal foods, including fish and seafood, but also bean products, sweets, desserts, and fried foods. Our innovative approach to assembling 93 food items into 12 food groups based on the similarity of foods in terms of nutrient content, we first identified a dietary pattern that combines not only purine-increasing food components but also sweets, desserts, and fried foods. Our study showed sweets and desserts were also components of the dietary pattern, which was associated with increased risk of hyperuricemia. Previous studies found either sweet dietary patterns (32) or sugar-containing carbonated beverages (33) or fructose (34) are associated with an increased risk of high uric acid levels.

Limited studies have observed that fried foods are linked with an increased level of SUA. Similar to our findings, a case–control study found that animal products and a fried-food dietary pattern, especially fried wheat products, were associated with a higher risk of hyperuricemia (18). A higher intake of fried foods has adverse cardiometabolic health effects, possibly via increasing blood pressure, and leads to central obesity and disorder in lipid metabolism (35). Therefore, it is recommended to conduct clinical and animal research to evaluate the precise physiology behind the role of fried food in metabolic and liver disease. The increased risk could be due to higher trans-fatty acid or purine content in the fried food, which affects purine metabolism or increases the risk of metabolic disorders, such as central obesity, high blood pressure, and lipid disorders. Studies showed that these conditions have a bidirectional association with elevated SUA levels (36). However, extensive epidemiological and experimental studies have yet to be conducted to confirm the association between fried foods and the risk of hyperuricemia, suggesting the need for such studies to unveil the underlying mechanism.

In subgroup analysis, the association between hyperuricemia and a dietary pattern characterized by high protein, along with sweets, desserts, and fried foods, was only significant among participants under 60 years of age. The exact mechanism by which this diet affects only people in this age group is a topic for further investigation. It could be due to metabolic differences or lifestyle factors that make them more susceptible to the effects of certain dietary components, such as sweets, desserts, and fried food. Further research is needed to understand these mechanisms and to develop targeted diet recommendations for different age groups.

In addition, when we performed mediation analysis to estimate the mediating effects of obesity, LDL, and liver disease, the association between dietary patterns and hyperuricemia risk was significantly mediated by high BMI, in combination with either LDL and/or fatty liver. Despite the observed wide confidence interval due to the involvement of multiple model components, the lower bound still suggests a statistically significant mediation effect, supporting the relevance of the pathway. The possible explanation for obesity and LDL mediating the association might be due to the higher amount of saturated fat (mostly from meat), trans fat (mostly from fried food), fructose, and added sugar (mostly from pastries and desserts) in the dietary pattern, which have been observed to be strongly associated with dyslipidemia and obesity (37, 38). On the other hand, the dietary pattern’s adverse effect on the risk of hyperuricemia may be biologically linked to adiposity and liver function, suggesting the need for future clinical trials or experimental studies. Another possible reason might be the role of a fair amount of purine content in the food items loaded in the dietary pattern in purine metabolism.

### Study limitations

First, we used the FFQ method to collect food consumption information in the past year; therefore, recall bias may affect the accuracy of reported food intake. Second, we cannot rule out the possibility that unmeasured factors, primarily leading to residual error, might contribute to the observed association. Third, as the study was cross-sectional, having its inherent design drawbacks, the causality of the observed association in this study could not be established. Additionally, the study was conducted among residents in the Southeast coastal region of China with a specific diet culture that may not be generalizable to the whole Chinese population. Moreover, we excluded subjects with chronic diseases in the main analysis, which may not be representative of the general population. However, excluding the subjects with chronic diseases reduces the reverse causality problem to some extent.

## Conclusion

The present study identifies that a dietary pattern high in protein, fat, and sugar is associated with an increased risk of hyperuricemia, particularly in individuals below 60 years old. The association is significantly mediated through higher BMI combined with LDL and/or fatty liver. The current research suggests that moderating the consumption of animal foods and minimizing the intake of fried foods, sweets, and desserts may help prevent hyperuricemia.

## Data Availability

The datasets presented in this article are not readily available due to the privacy of the patients and confidentiality reasons. Requests to access the datasets should be directed to ywm@fjmu.edu.cn.

## Footnotes

^1^ https://cohort.fjmu.edu.cn/co-cas/login
