# Supplementary material for: Dietary patterns associated with hyperuricemia among the southeast coastal Chinese population
Source: Front Nutr. 2025 Oct 8;12:1670666. doi: 10.3389/fnut.2025.1670666 (PMC12540132; doi:10.3389/fnut.2025.1670666)
Supplement: Supplementary file 1 [file Table_1.DOCX]

**Supplementary Table 1. Food items in each food group.**

| **Food groups** | **Food names** |
| --- | --- |
| Staple foods | Rice, porridge, rice noodles or vermicelli, pasta (noodles), steamed bread, meat buns, vegetable buns, corn, dumplings, wontons or meat swallows, kompyang, packed instant noodles, multi-grain porridge, buns (sweet potato filling), buns (peanut filling), oatmeal |
| Tubers | Potatoes |
| Beans and legume products | Yellow beans, other dried beans, tofu, soybean milk, bean products, fried bean products, bean curd and bean paste |
| Seeds and nuts | Tree nuts, dried fruits, peanuts, peanut soup/paste |
| Milk and dairy products | Fresh/boxed milk, yogurt, milk powder |
| Eggs | Chicken eggs, duck eggs, quail eggs, goose eggs, salted eggs, preserved eggs |
| Fish and seafood | Freshwater fish, sea fish, squid or octopuses, shrimp, crabs, shellfish, jellyfish, sea tangle, laver, fish balls, dried fish/flatfish, dried shellfish(clam/oyster/scallop), salted fish |
| Meat | Pork, beef/lamb/rabbit, chicken/duck/goose, liver or chicken heart, other viscera, processed meats |
| Fruits | Apples, pears, peaches, plums, winter/green jujube, grapes and raisins, strawberries, citrus/oranges/grapefruits, guavas, pineapples, longan, lychees, mangoes, bananas, papayas, loquats, pitaya, watermelons, cantaloupe, musk melon, ginseng fruit, kiwi fruit |
| Vegetables | Legumes/fresh beans, solanaceae fruits, melon vegetables, onions and garlic, dark vegetables, light-colored vegetables, water vegetables, fungi, pickled vegetables |
| Sweets and desserts | Western pastries, cookies, candy/compote/chocolate, ice cream |
| Fried foods | Fried pasta, fried potatoes (chips) |

**Supplementary Table 2. Four prominent components with eigenvector loadings of major food groups obtained by principal component analysis with varimax rotation**

| **Food Group** | **RC1** | **RC2** | **RC3** | **RC4** |
| --- | --- | --- | --- | --- |
| **Staple foods** | -0.19 | -0.27 | **-0.35** | **-0.60** |
| **Tubers** | **0.68** | -0.19 | -0.28 | -0.21 |
| **Bean and legume products** | 0.13 | **0.48** | 0.02 | 0.05 |
| **Seeds and nuts** | 0.01 | -0.10 | -0.20 | **0.86** |
| **Milk and dairy products** | 0.07 | 0.17 | **0.55** | -0.11 |
| **Eggs** | -0.08 | -0.17 | **0.71** | 0.05 |
| **Fish and seafood** | **0.32** | **0.45** | 0.08 | 0.15 |
| **Meat** | -0.06 | **0.53** | 0.29 | 0.17 |
| **Fruits** | **0.42** | 0.19 | **0.42** | 0.17 |
| **Vegetables** | **0.64** | 0.16 | 0.15 | 0.20 |
| **Sweets and desserts** | 0.07 | **0.50** | 0.08 | -0.09 |
| **Fried foods** | -0.16 | **0.63** | -0.18 | -0.02 |

RC: rotated component derived from principal component analysis

Proportion variance: RC1 = 10%; RC2 = 13%; RC3 = 12%; RC4 = 11%

Bold values are loadings of food groups > |0.30| in the components

**Supplementary Table 3. Comparing the effect on hyperuricemia risk with dietary components using the linear and non-linear models**

| **Model** | **Component** | **Coefficient** | **S.E.** | **Wald Z** | **P value** |
| --- | --- | --- | --- | --- | --- |
| **Linear** |  |  |  |  |  |
|  | RC1 | 0.0038 | 0.0361 | 0.11 | 0.9161 |
|  | RC2 | 0.0969 | 0.0383 | 2.53 | 0.0114 |
|  | RC3 | 0.0129 | 0.0371 | 0.35 | 0.7272 |
|  | RC4 | 0.0041 | 0.0362 | 0.11 | 0.909 |
| **Non-linear** |  |  |  |  |  |
|  | RC1 | -0.1264 | 0.1567 | -0.81 | 0.4202 |
|  | RC1' | 0.3971 | 0.6629 | 0.6 | 0.5491 |
|  | RC1'' | -0.9366 | 1.9816 | -0.47 | 0.6365 |
|  | RC2 | -0.0112 | 0.1698 | -0.07 | 0.9474 |
|  | RC2' | 0.5453 | 0.8924 | 0.61 | 0.5411 |
|  | RC2'' | -1.415 | 2.4704 | -0.57 | 0.5668 |
|  | RC3 | 0.2084 | 0.1496 | 1.39 | 0.1636 |
|  | RC3' | -0.6877 | 0.5257 | -1.31 | 0.1908 |
|  | RC3'' | 2.072 | 1.6722 | 1.24 | 0.2153 |
|  | RC4 | -0.0868 | 0.1492 | -0.58 | 0.5607 |
|  | RC4' | 0.325 | 0.5741 | 0.57 | 0.5713 |
|  | RC4'' | -1.0975 | 2.204 | -0.5 | 0.6185 |

RC: Rotated principal component; S.E: Standard error

The adjusted model accounted for age, sex, body mass index, fatty liver disease, estimated glomerular filtration rate, alcohol user and smoking status, low-density lipoprotein, high-density lipoprotein, triglyceride, systolic blood pressure, diastolic blood pressure, fasting glucose, glycated hemoglobulin A1c, physical activity, total energy intake per day, and other rotated components (RC1, RC2, RC3, and RC4).

RC1: The first component had a high intake of vegetables, tubers, fruits, fish, and seafood.

RC2: The second component was characterized by a high intake of meat, fish and seafood, bean products, sweets, desserts, and fried food.

RC3: The third component was characterized by a lower intake of staple food and higher consumption of milk and dairy products, eggs, and fruits.

RC4: The Fourth pattern had a notably high intake of nuts and a low intake of staple foods.

**Supplementary Table 4. Effect of each dietary component on hyperuricemia risk compared low vs. high using the linear and non-linear models**

| **Model** | **Component** | **Effect** | **Low** | **High** | **Diff.** | **Effect** | **S.E.** | **Lower 0.95** | **Upper 0.95** |
| --- | --- | --- | --- | --- | --- | --- | --- | --- | --- |
| **Linear** |  |  |  |  |  |  |  |  |  |
|  | **RC1** | Beta | -0.6796 | 0.5376 | 1.2173 | 0.0046 | 0.0439 | -0.0815 | 0.0908 |
|  |  | Odds Ratio | -0.6796 | 0.5376 | 1.2173 | 1.0046 | NA | 0.9217 | 1.0950 |
|  | **RC2** | Beta | -0.6512 | 0.4527 | 1.1039 | 0.1070 | 0.0423 | 0.0241 | 0.1898 |
|  |  | Odds Ratio | -0.6512 | 0.4527 | 1.1039 | 1.1129 | NA | 1.0244 | 1.2090 |
|  | **RC3** | Beta | -0.6648 | 0.5468 | 1.2116 | 0.0157 | 0.0449 | -0.0724 | 0.1037 |
|  |  | Odds Ratio | -0.6648 | 0.5468 | 1.2116 | 1.0158 | NA | 0.9302 | 1.1093 |
|  | **RC4** | Beta | -0.5792 | 0.4087 | 0.9879 | 0.0041 | 0.0358 | -0.0661 | 0.0742 |
|  |  | Odds Ratio | -0.5792 | 0.4087 | 0.9879 | 1.0041 | NA | 0.9361 | 1.0771 |
| **Non-linear** | |  |  |  |  |  |  |  |  |
|  | **RC1** | Beta | -0.6796 | 0.5376 | 1.2173 | 0.0124 | 0.0916 | -0.1671 | 0.1918 |
|  |  | Odds Ratio | -0.6796 | 0.5376 | 1.2173 | 1.0124 | NA | 0.8461 | 1.2115 |
|  | **RC2** | Beta | -0.6512 | 0.4527 | 1.1039 | 0.1356 | 0.0900 | -0.0408 | 0.3120 |
|  |  | Odds Ratio | -0.6512 | 0.4527 | 1.1039 | 1.1452 | NA | 0.9600 | 1.3661 |
|  | **RC3** | Beta | -0.6648 | 0.5468 | 1.2116 | -0.0585 | 0.0934 | -0.2416 | 0.1245 |
|  |  | Odds Ratio | -0.6648 | 0.5468 | 1.2116 | 0.9432 | NA | 0.7854 | 1.1326 |
|  | **RC4** | Beta | -0.5792 | 0.4087 | 0.9879 | 0.0325 | 0.0867 | -0.1375 | 0.2024 |
|  |  | Odds Ratio | -0.5792 | 0.4087 | 0.9879 | 1.0330 | NA | 0.8716 | 1.2244 |

RC: Rotated principal component; S.E: Standard error

The adjusted model accounted for age, sex, body mass index, fatty liver disease, estimated glomerular filtration rate, alcohol user and smoking status, low-density lipoprotein, high-density lipoprotein, triglyceride, systolic blood pressure, diastolic blood pressure, fasting glucose, glycated hemoglobulin A1c, physical activity, total energy intake per day, and other rotated components (RC1, RC2, RC3, and RC4).

RC1: The first component had a high intake of vegetables, tubers, fruits, fish, and seafood.

RC2: The second component was characterized by a high intake of meat, fish and seafood, bean products, sweets, desserts, and fried food.

RC3: The third component was characterized by a lower intake of staple food and higher consumption of milk and dairy products, eggs, and fruits.

RC4: The Fourth pattern had a notably high intake of nuts and a low intake of staple foods.

**Supplementary Table 5. Comparing the p-value for the combined and the non-linear component of the non-linear model**

| **Factor** | **Chi-Square** | **D.F.** | **P** |
| --- | --- | --- | --- |
| RC1 | 1.05 | 3 | 0.7893 |
| Nonlinear | 1.03 | 2 | 0.5964 |
| RC2 | 7.04 | 3 | 0.0706 |
| Nonlinear | 0.45 | 2 | 0.7999 |
| RC3 | 1.99 | 3 | 0.5739 |
| Nonlinear | 1.8 | 2 | 0.406 |
| RC4 | 0.51 | 3 | 0.9171 |
| Nonlinear | 0.45 | 2 | 0.7994 |

RC: Rotated principal component; D.F.: Degree of freedom

The adjusted model accounted for age, sex, body mass index, fatty liver disease, estimated glomerular filtration rate, alcohol user and smoking status, low-density lipoprotein, high-density lipoprotein, triglyceride, systolic blood pressure, diastolic blood pressure, fasting glucose, glycated hemoglobulin A1c, physical activity, total energy intake per day, and other rotated components (RC1, RC2, RC3, and RC4).

RC1: The first component had a high intake of vegetables, tubers, fruits, fish, and seafood.

RC2: The second component was characterized by a high intake of meat, fish and seafood, bean products, sweets, desserts, and fried food.

RC3: The third component was characterized by a lower intake of staple food and higher consumption of milk and dairy products, eggs, and fruits.

RC4: The Fourth pattern had a notably high intake of nuts and a low intake of staple foods.

RC4: The Fourth pattern had a notably high intake of nuts and a low intake of staple foods.


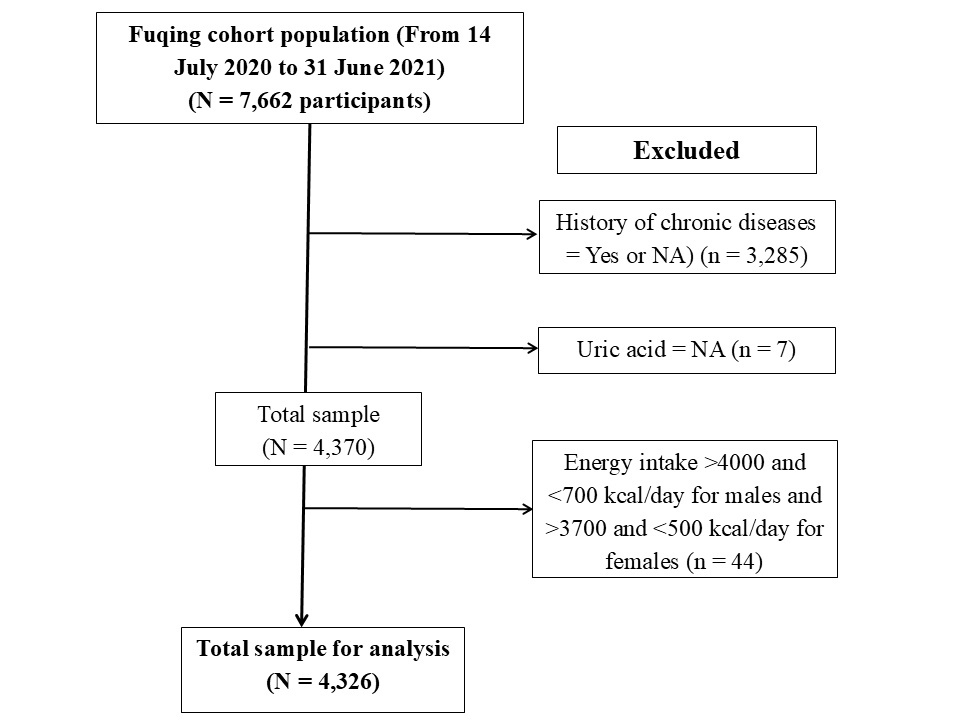


**Supplementary Figure 1. Participant flow chart to select the study population for the present analysis.** The figure describes the total sample participants after excluding the individuals with chronic disease conditions, unreliable energy intake, and missing values of serum uric acid.


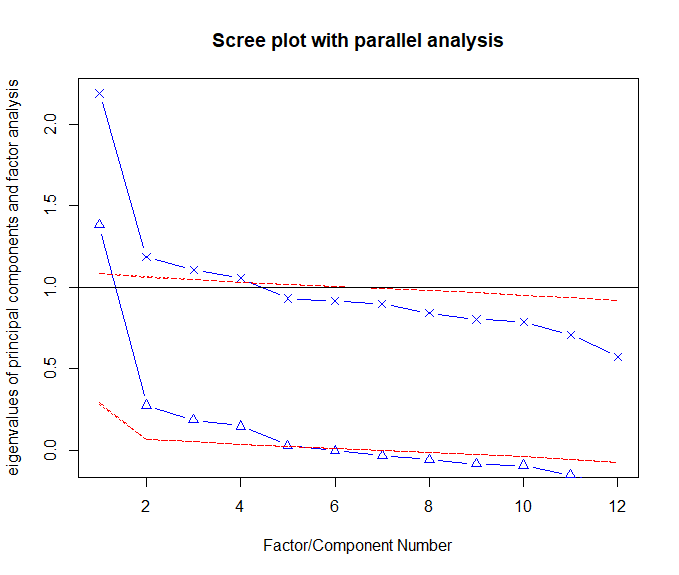


**Supplementary Figure 2. Scree plot with parallel analysis to determine the number of principal components.** The figure shows the eigenvalues of principal components on the y-axis and the component numbers on the x-axis, with a red line indicating the threshold for retaining the number of components via parallel analysis and elbow techniques. This technique identifies a total of 4 potential components for further analysis of dietary pattern identification.

**Supplementary Figure 3. Comparing the effect of each dietary pattern in linear and non-linear models**


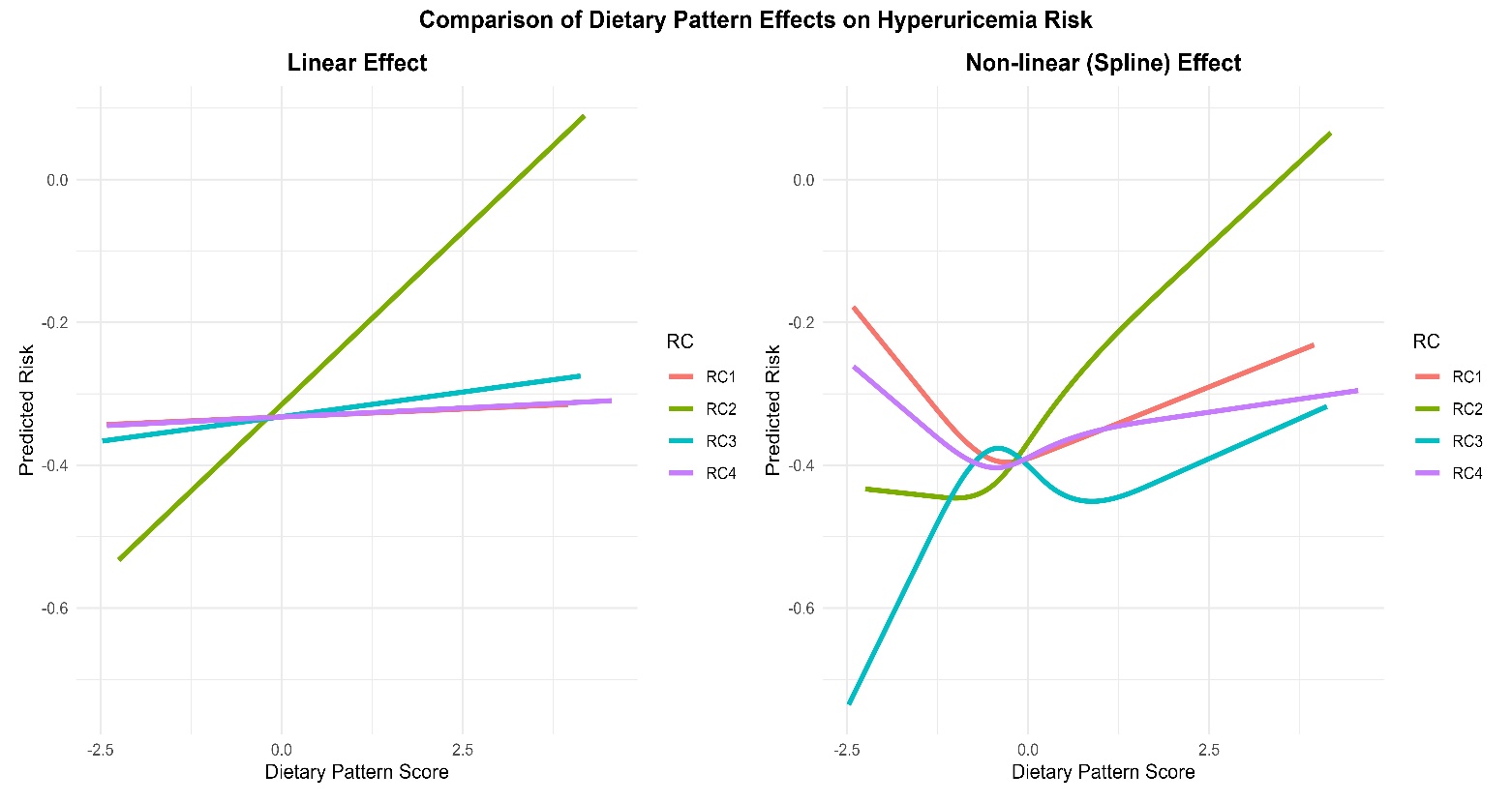


RC: Rotated principal component

The adjusted model accounted for age, sex, body mass index, fatty liver disease, estimated glomerular filtration rate, alcohol user and smoking status, low-density lipoprotein, high-density lipoprotein, triglyceride, systolic blood pressure, diastolic blood pressure, fasting glucose, glycated hemoglobulin A1c, physical activity, total energy intake per day, and other rotated components (RC1, RC2, RC3, and RC4).

RC1: The first component had a high intake of vegetables, tubers, fruits, fish, and seafood.

RC2: The second component was characterized by a high intake of meat, fish and seafood, bean products, sweets, desserts, and fried food.

RC3: The third component was characterized by a lower intake of staple food and higher consumption of milk and dairy products, eggs, and fruits.

RC4: The Fourth pattern had a notably high intake of nuts and a low intake of staple foods.

RC4: The Fourth pattern had a notably high intake of nuts and a low intake of staple foods.
